# A landmark in drug discovery based on complex natural product synthesis

**DOI:** 10.1038/s41598-019-45001-9

**Published:** 2019-06-17

**Authors:** Satoshi Kawano, Ken Ito, Kenzo Yahata, Kazunobu Kira, Takanori Abe, Tsuyoshi Akagi, Makoto Asano, Kentaro Iso, Yuki Sato, Fumiyoshi Matsuura, Isao Ohashi, Yasunobu Matsumoto, Minetaka Isomura, Takeo Sasaki, Takashi Fukuyama, Yusuke Miyashita, Yosuke Kaburagi, Akira Yokoi, Osamu Asano, Takashi Owa, Yoshito Kishi

**Affiliations:** 1 Eisai Co., Ltd., Tokodai, Tsukuba-shi, Ibaraki, Japan; 2 Harvard University, Cambridge, Massachusetts USA; 3 Eisai Co., Ltd., Koishikawa, Bunkyo-ku, Tokyo, Japan; 4 Eisai Co., Ltd., Sunayama, Kamisu-shi, Ibaraki, Japan; 5 Eisai Inc., Woodcliff Lake, New Jersey, USA

**Keywords:** Cancer, Organic chemistry, Molecular medicine

## Abstract

Despite their outstanding antitumour activity in mice, the limited supply from the natural sources has prevented drug discovery/development based on intact halichondrins. We achieved a total synthesis of C52-halichondrin-B amine (E7130) on a >10 g scale with >99.8% purity under GMP conditions. Interestingly, E7130 not only is a novel microtubule dynamics inhibitor but can also increase intratumoural CD31-positive endothelial cells and reduce α-SMA-positive cancer-associated fibroblasts at pharmacologically relevant compound concentrations. According to these unique effects, E7130 significantly augment the effect of antitumour treatments in mouse models and is currently in a clinical trial. Overall, our work demonstrates that a total synthesis can address the issue of limited material supply in drug discovery/development even for the cases of complex natural products.

## Introduction

Halichondrins are structurally complex natural products isolated from various marine sponges^1–8^. Halichondrins are known to exhibit exceptional *in vivo* antitumour activities in mice in addition to excellent *in vitro* activities towards a variety of human cancer cell types. These compounds are novel inhibitors of microtubule dynamics, but their mode of action is distinctly different from those of other microtubule-targeted drugs such as paclitaxel^9–11^. Halichondrin B was shown to be more effective than vinblastine, another type of microtubule-targeted drug, in the LOX melanoma bone marrow metastasis model in nude rats, while no difference in antitumour activity was observed in the subcutaneous (s.c.) xenograft model with the same melanoma cell line^12^. Despite their extraordinarily potent and unique anticancer activities, to date, intact halichondrins have not been studied as anticancer drugs in humans because the material could not be secured via either isolation from natural sources or chemical synthesis^13^. Nonetheless, its exceptional *in vivo* antitumour activity in mouse xenograft models may suggest that halichondrins are not simply microtubule-targeted drugs, thereby having encouraged us to undertake drug development efforts using intact halichondrins.

## Results

### Total synthesis of a direct derivative of intact halichondrins

**Figure 1:**
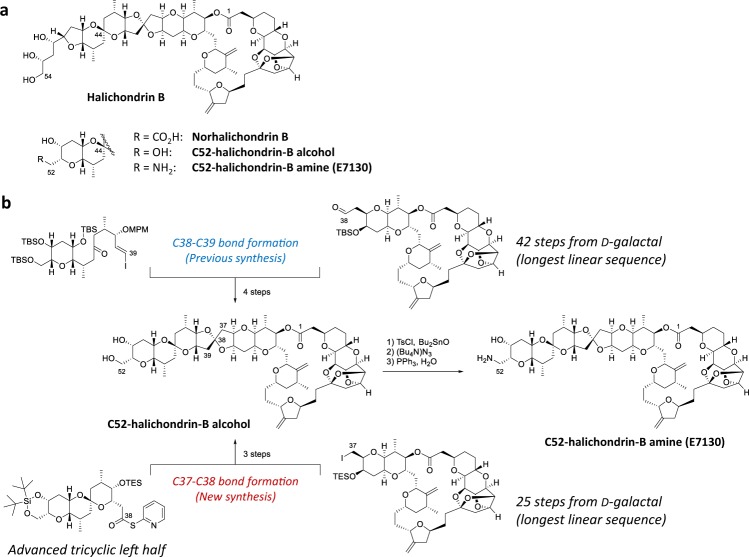
Chemical structures referenced in this work and the scale-up strategy. (**a**) Chemical structures of halichondrin B, norhalichondrin B, C52-halichondrin-B alcohol, and C52-halichondrin-B amine/E7130. (**b**) The strategy for scale-up. The previous synthesis relied on a C38-C39 bond formation strategy. The new synthesis relies on a C37-C38 bond formation strategy. The latter provided 19.5 g of C52-halichondrin-B alcohol with 99.84% purity. Fifteen grams of this compound was used to synthesize 11.5 g of E7130 with 99.81% purity as the first GMP batch (92 overall steps).

Specifically, we recognized that our recent studies in the total syntheses of halichondrins could solve the problem of limited material supply via chemical synthesis. This approach has led us to E7130, as a promising anticancer drug candidate (Fig. 1a). The first batch of E7130 was prepared on a milligram scale via a modified version of the original synthesis^13^. This synthesis required 109 steps from commercially available materials, and HPLC purification of E7130 was essential to remove a number of unidentified impurities. Meanwhile, significant progress has been made in the total synthesis of halichondrins, including the discovery and development of catalytic, asymmetric Ni/Cr-mediated cross-coupling reactions and Zr/Ni-mediated one-pot ketone synthesis. These developments have been incorporated to this project. Thus, C52-halichondrin-B alcohol (E7130 precursor) was synthesized from the left and right halves via a C37-C38 bond formation in the new synthesis^14–16^, whereas the previous synthesis used a C38-C39 bond formation as a key step^13^ (Fig. 1b, and the relevant parts of E7130 synthesis are paragraph numbers [00868]-[00909] in WO2019/010363 and paragraph numbers [00387]-[00417], [00428]-[00432] and [00544]-[00547] in WO2016/176560). This modification resulted in remarkable improvements on the overall efficiency of synthesis in several respects. First, the longest linear sequence of the right half was reduced from 42 steps to 25 steps from a commercially available material. Second, in the new synthesis, the [6,6]-spiroketal in the left half was built before the final coupling, whereas it was constructed via an oxy-Michael reaction after the final coupling in the previous synthesis. Although both routes gave the desired product as the major product, the isolated yield of C52-halichondrin-B alcohol in the new route was dramatically higher than that in the original route. Third, a practical and scalable synthesis was developed by selecting the appropriate coupling conditions for four catalytic, asymmetric Ni/Cr-mediated coupling reactions as well as for other transformations^17^. Overall, we successfully synthesized 19.5 g of C52-halichondrin-B alcohol with 99.84% purity via a total synthesis^14^. From 15.0 g of this material, we obtained 11.5 g of E7130 with 99.81% purity, and the material was isolated by reverse-phase medium-pressure chromatography (Fig. 1b).

### E7130 increases intratumoural microvessel density

**Figure 2:**
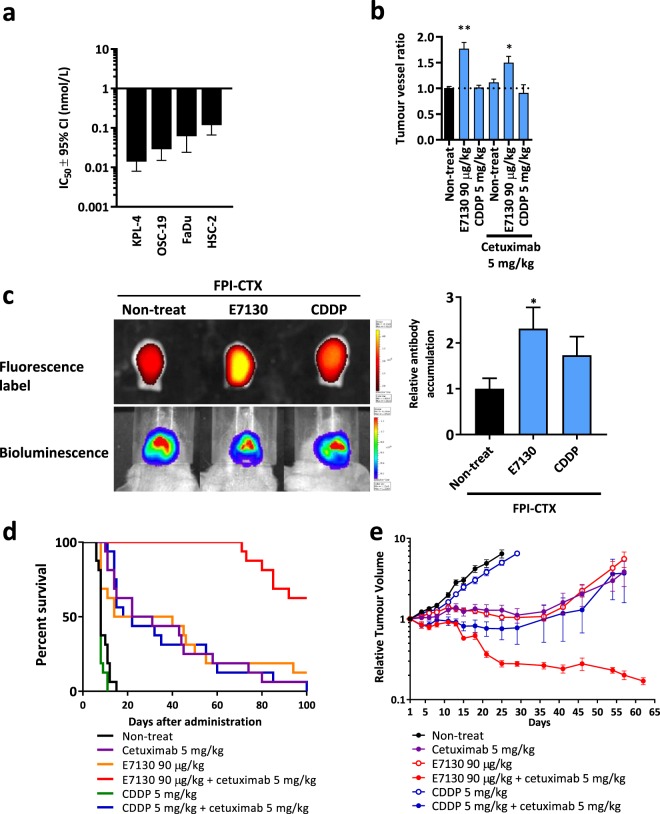
Biochemical, cellular, and *in vivo* mechanistic activity of E7130. (**a)** The effect of E7130 on the viability of 4 cell lines after 3 days represented as the concentration of E7130 required to decrease cell viability to 50% (IC_50_) and 95% confidence interval (CI). (**b)** HSC-2 squamous cell carcinoma of the head and neck orthotopically transplanted tumours were collected 4 days after the administration. Data show the mean tumour vessel ratios of treated to non-treated ± s.e.m. (n = 3). *P = 0.0228, **P = 0.0030 versus non-treated (Dunnett’s multiple comparison test). (**c)** The accumulation of fluorescent-labelled cetuximab (FPI-CTX) was analysed using an *In Vivo* Imaging System (IVIS) 5 days after the indicated administration. Representative *in vivo* bioluminescence images and *ex vivo* fluorescence labelling in resected tongues are shown. The values of total flux (photon/second) were normalized with each bioluminescent value (photon/second). The graph shows the mean FPI-CTX accumulation ratios to the accumulation in the FPI-CTX mono-administration group ± s.e.m. (n = 4). *P = 0.0440 versus FPI-CTX mono-administration (Two-tailed unpaired *t* test). (**d)** Effect of the indicated administration on day 1, day 8, and day 15 on survival in the HSC-2 orthotopic transplantation mouse model (n = 16). (**e)** Effect of the indicated administration on day 1, day 8, and day 15 on the relative tumour volume of the subcutaneous HSC-2 subcutaneous xenograft model. In this study, nine days after the cell inoculation subcutaneously in the right flank of Balb/C-nu mice, 36 mice were selected based on their tumour volumes and shapes of tumours, and were randomly allocated into 6 groups (day 1). The mean tumour volume of mice assigned to the groups on day 1 were 321.6 mm^3^. The mean relative tumour volume to the tumour volume on day 1 ± s.e.m. is shown (n = 6).

With totally synthetic E7130 in hand, we began to study *in vivo* antitumour activity in mice reported for the halichondrin class of marine natural products. E7130 showed inhibitory activity towards tubulin polymerization in the cell-free system and caused the disappearance of the EB3 comet-structures, indicating that E7130 has the ability to suppress microtubule dynamics (Supplementary Fig. S1a,b, Supplementary Video 1 and 2). E7130 exhibited potent antiproliferative activities against several cancer cell lines *in vitro* with subnanomolar IC_50_ values (Fig. 2a), which shows a more than 10-fold different in potency of E7130 across the cell lines. Although the reason of this has not been clarified, sensitivity to microtubule-targeted drugs is known to be affected by the expression of the drug efflux pump such as P-glycoprotein and mutations in and/or alteration of tubulin isotype levels^18,19^. E7130 also exerted significant and dose-dependent antitumour activities with tumour regression in KPL-4 HER2-positive breast cancer and OSC-19 squamous cell carcinoma of the head and neck (SCCHN) s.c. xenograft models (Supplementary Fig. S1c,d). In addition to its direct antitumour activities, we noted that E7130 increased the intratumoural microvessel density (MVD) in xenografts (Fig. 2b). To investigate whether the increased intratumoural microvessels are functional, we assessed the accumulation of cetuximab (CTX) in the presence of E7130 in the HSC-2 SCCHN orthotopic transplantation mouse model. Indeed, we found that E7130 significantly enhanced delivery of CTX into tumours (Fig. 2c). Moreover, notable tumour regression was observed when E7130 was used in combination with CTX. On the other hand, cisplatin (CDDP), which did not increase intratumoural MVD nor delivery of CTX, did not show tumour regression activity, even when used in combination with E7130 (Supplementary Fig. S2). The synergistic effect was also demonstrated by the apparent prolongation of survival in the model, and the prominent antitumour activity of the compounds together was also confirmed in the HSC-2 s.c. xenograft model (Fig. 2d,e). These data unequivocally supported that the increased intratumoural MVD caused by E7130 enhanced the delivery of CTX into tumours and led to a clear tumour regression and survival advantage. This mechanism of action is further supported by the data from two other cell line s.c. inoculation models. The first is the KPL-4 model, in which E7130 increased intratumoural MVD (Supplementary Fig. S3d). E7130 in combination with trastuzumab showed a stronger antitumour activity than each monotherapy in this model (Supplementary Fig. S3b,c). The second is the CT26 murine colon carcinoma model, in which E7130 in combination with an anti-mouse PD-1 antibody increased intratumoural accumulation of the antibody. The combination of these compounds delayed tumour growth relative to each monotherapy (Supplementary Fig. S4).

### E7130 ameliorates the tumour microenvironment to improve cancer treatment

**Figure 3:**
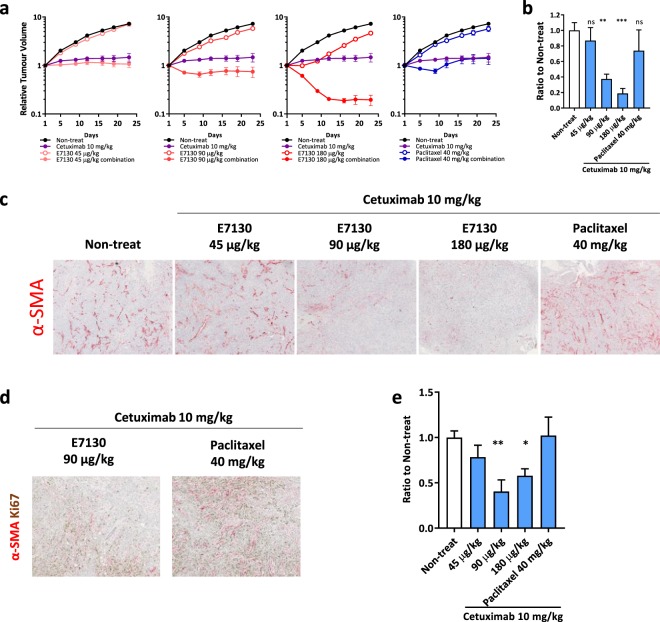
E7130 showed an anti-CAF effect leading to combinational effect against FaDu xenografts. (**a)** Effect of the indicated administration on day 1 on the relative tumour volume of FaDu subcutaneous xenografts. The mean relative tumour volume to the volume on day 1 ± s.e.m. is shown (n = 6). (**b–e**) FaDu xenografts were collected 10 days after the indicated administration. The areas of α-SMA (**b–d**), Ki67 (**d**), and tenascin-C (**e**) were analysed by immunohistochemistry. The data shown are the mean area of α-SMA-positive CAFs (**b**) or tenascin-C (**e**) to those of the non-treated group ± s.e.m. (n = 5). (**b**) **P = 0.0021, ***P = 0.0002, (**e**) *P = 0.0360, **P = 0.0031 versus the non-treated group (Dunnett’s multiple comparison test). Representative images are shown in (**c**,**d)**.

We further analysed the combined effect of E7130 with CTX in the FaDu SCCHN s.c. xenograft model. As in the case of the HSC-2 model, a remarkable combined effect was observed above the dose of 90 μg/kg, one-half of the maximum tolerated dose in mice, even after a single administration. Interestingly, tumour regression in the E7130 combination groups (90 μg/kg and 180 μg/kg) was sustained for a longer period than was seen in the paclitaxel combination group (Fig. 3a). These observations led us to the hypothesis that an additional function of E7130 inhibits tumour regrowth in the combinational groups. To test this hypothesis, we analysed the changes in the intratumoural cancer-associated fibroblasts (CAFs) in the xenografts because intratumoural CAFs are key characteristics of SCCHN and are negative prognostic factors contributing to worse clinical outcomes for those with the disease^20,21^. The effect on CAFs was analysed by quantifying the area of α-SMA-positive cells, which is a well-known activated CAF marker in SCCHN^22^, and the immunohistochemical analyses were performed with tumour tissues collected 10 days after the administration, which was just before the tumour regrowth in the paclitaxel combination group. Interestingly, E7130 in the combination treatment significantly reduced the α-SMA-positive CAFs at 90 μg/kg and 180 μg/kg in the xenografts, whereas paclitaxel did not show this effect (Fig. 3b,c). In contrast, ER-TR7 (pan-fibroblast marker) staining clearly indicated that the E7130 combination did not reduce the overall stroma area (Supplementary Fig. S5a), which suggests that E7130 specifically reduced the α-SMA-positive CAFs, and the E7130 combination modulated the phenotypes of the fibroblasts. Furthermore, Ki67-positive cancer cells were observed adjacent to the α-SMA-positive CAFs in tumours treated with the paclitaxel combination, whereas such staining patterns were much less frequently observed in the E7130 combination (Fig. 3d). As the candidate for the downstream effector in α-SMA-positive CAFs, which can account for its tumour-promoting function, we examined the expression of tenascin-C and EDA-fibronectin, extracellular matrix (ECM) proteins^23,24^. Immunohistochemical analysis demonstrated that tenascin-C and EDA-fibronectin were reduced by E7130 in combination with CTX but not by paclitaxel in combination with CTX (Fig. 3e, Supplementary Fig. S5b–d). These results strongly suggested that α-SMA-positive CAFs provide some growth-promoting signals to neighbouring cancer cells, which led to tumour regrowth, and the difference in the potencies of the treatments for reducing such tumour-promoting α-SMA-positive CAFs can account for their different antitumour activities in terms of the suppression of regrowth between treatment with E7130 and CTX and treatment with paclitaxel and CTX. In addition, we confirmed the potency of E7130 monotherapy for reducing α-SMA-positive CAFs in this model and the other types of SCCHN xenografts (Supplementary Fig. S5e–h).

### E7130 impedes the TGF-β-induced myofibroblast transdifferentiation process

**Figure 4:**
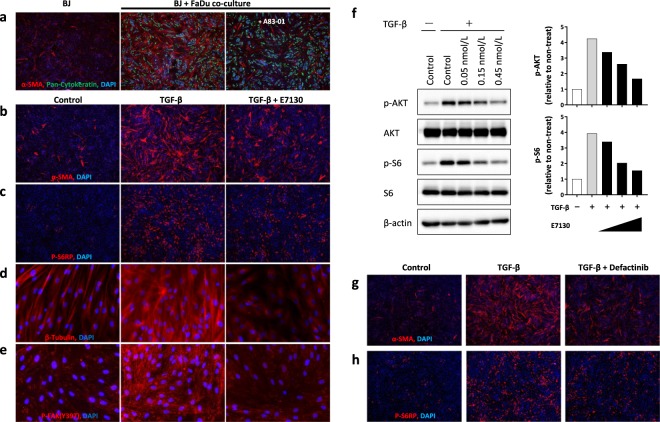
E7130 reduced TGF-β-induced α-SMA expression in the *in vitro* CAF-inducing system. (**a**) BJ cells (normal human lung fibroblasts) were co-cultured with FaDu cells for three days in the absence (vehicle) or presence of A83-01, a potent selective TGF-β-R inhibitor, and the expression of α-SMA was analysed by immunofluorescence staining (red colour). Samples were also stained with anti-pan-human cytokeratin (green colour) for cancer cell staining and DAPI (blue colour) for nuclear staining. b-f, BJ cells were treated with E7130 (0.15 nmol/L in immunofluorescence staining data and indicated concentrations in western blot analysis data) and TGF-β (1 ng/mL) for 2 days. (**b**–**e**) Samples were stained with the indicated antibody (red colour) and DAPI (blue colour) for nuclear staining. (**f)** Western blot images and quantification of the images. The graph shows ratios of the results of treatment groups to the non-treated group. (**g**,**h)** BJ cells were pretreated with defactinib (1 μmol/L) and TGF-β (1 ng/mL) for 2 days. The lens magnification used was ×4 (**a**–**c**,**g**,**h**) or ×40 (**d**,**e**).

Next, we analysed the molecular mechanisms of the α-SMA-positive CAF reduction using an *in vitro* culture system. We first confirmed that the expression of α-SMA was induced in BJ normal human fibroblasts upon co-cultivation with FaDu cells, and the expression was attenuated by treatment with A83-01, a potent selective inhibitor of the TGF-β-receptor (Fig. 4a). These results suggested that TGF-β plays a major role as a mediator of α-SMA induction in this system. In addition, immunofluorescence analysis revealed that treatment with E7130 interfered with α-SMA induction by TGF-β in BJ cells without growth inhibitory activity (Fig. 4b, Supplementary Fig. S6a). We further found that E7130 did not significantly change the TGF-β-induced phosphorylation and nuclear localization of Smad2/3 (Supplementary Fig. S6d,e), but it reduced the activation of the PI3K/AKT/mTOR pathway, which plays essential roles in TGF-β-induced α-SMA expression (Fig. 4c,f and Supplementary Fig. S6b,c). Moreover, TGF-β treatment enhanced β-tubulin expression and microtubule network formation, which were diminished by co-treatment with E7130 in BJ cells (Fig. 4d). Under the same experimental settings, the enhanced formation of focal adhesions, which were detected as punctate structures with the antibody against phosphorylated FAK at tyrosine-397, after stimulation with TGF-β was decreased in BJ cells by treatment with E7130 (Fig. 4e). Many signalling complexes are assembled in focal adhesion sites, and those complexes dispatch several downstream signals, including those involved in the PI3K/AKT/mTOR pathway^25^. In fact, treatment with defactinib, an FAK inhibitor, clearly decreased the level of α-SMA expression as well as S6 ribosomal protein phosphorylation induced by TGF-β in BJ cells (Fig. 4g,h). We confirmed that all of the abovementioned phenomena were also observed in other normal human fibroblasts (TIG3 cells) (Supplementary Fig. S7). Considering that several reports have also found that plus ends of microtubules interact with focal adhesion sites and modulate their functions in interphase cells^26^, our results strongly suggest that E7130 impedes the TGF-β-induced myofibroblast transdifferentiation process by disrupting microtubule network formation, which is important for focal adhesion assembly and thereby the downstream activation of the PI3K/AKT/mTOR pathway.

## Discussion

As mentioned, we successfully synthesized >10 g of E7130 with >99.8%-purity under good manufacturing practice (GMP) conditions, which provides us the opportunity to conduct a number of preclinical evaluations. We also note that >10 g of E7130 prepared under GMP conditions would be sufficient for the clinical studies planned with projected human doses based on the preclinical studies. E7130 was well tolerated in animal studies at the doses and schedules examined as indicated by the little to no decrease in relative body weight (<20% change) and negligible changes in behaviour (Supplementary Figs S1c,d, S3c,e and S4d). In particular, a dose of 90 μg/kg, one-half of the maximum tolerated dose in mice, showed a prominent combinational effect with the other drug (Figs 2d,e and 3a, Supplementary Figs S3b and S4e), which suggests that E7130 may have a different mechanism than other microtubule-targeted drugs. In fact, although there are many compounds that show cytotoxic effects against tumours by inhibiting tubulin assembly^27^, a microtubule-targeted drug with both tumour-vascular remodelling activity and an anti-CAF effect has not been reported. Here, we prove that E7130 provides a means of ameliorating the tumour microenvironment to improve cancer treatment when used in combination with other compounds. Our data provide compelling evidence that the development of E7130 as a molecular-targeting agent is a viable strategy (Supplementary Fig. S8).

It is well known that fibroblasts can be activated to be CAF in the process of myofibroblast transdifferentiation followed by the upregulation of expression status of α-SMA^28^. This type of CAF, Type II polarized fibroblasts expressing α-SMA is reported to contribute to angiogenesis by releasing pro-angiogenic factors^29^. In consistent with this, a correlation between microvessel and α-SMA positive CAFs has been reported in head and neck cancers^30^. We, however, observed that E7130 increased intratumoural MVD while α-SMA positive CAFs were reduced in the same xenograft model (Fig. 2b and Supplementary Fig. S5g), which was different from the reported observations. It suggested that E7130 affect MVD and CAF independently. We would like to emphasize that E7130 impedes the myofibroblast transdifferentiation process also *in vitro* settings in which a factor of vascular status was avoided (Fig. 4, Supplementary Figs S6 and S7), and the result suggests that E7130 has direct effect against CAFs.

Considering future clinical strategies, we will continue our studies to further elucidate the molecular mechanism of actions of E7130. E7130 is currently in the Phase I trial in Japan at the starting dose of 25 μg/m^2^ on day 1 and day 15 of every 28-day cycle. We plan to first aim at a monotherapy for rare cancers because E7130 is presumed to be especially effective in diseases characterized by CAF abundance. For instance, neoplasm infiltrating between collagen bundles of the tumour is correlated with worse clinical outcomes in angiosarcomas^31^, suggesting that the anti-CAF effect of E7130 would improve the efficacy against the disease and fulfil the unmet medical needs.

In summary, a total synthesis has secured an access even to a complex natural product such as halichondrin, thereby providing us with new opportunities to identify potential drug candidate(s). In this project, based on their outstanding *in vivo* antitumour activity in mice, in conjunction with the study on experimental melanoma bone metastasis reported in 1996, we hypothesized that halichondrins are not simple microtubule-targeted compounds, leading us to the discovery of E7130, which harbors unique tumour microenvironment ameliorating effects. We specifically note that diligent research efforts over the past two decades have resulted in a revolution in the total synthesis of halichondrin, enabling us to address the unanswered biological questions relating to the tumour microenvironment effects of these compounds and further tackle the unmet medical needs of rare cancers.

## Methods

### Cell models

FaDu (ATCC) and CT26 (ATCC) cells were cultured in RPMI-1640 medium, HSC-2 (RIKEN) cells were cultured in EMEM, KPL-4 cells (Kawasaki Medical School) were cultured in DMEM, and OSC-19 (JCRB) cells were cultured in DMEM/F12. Each culture medium was supplemented with penicillin, streptomycin and 10% FBS. Cell line identities were confirmed by STR fingerprinting except murine cell line CT26, and all were found to be negative for mycoplasma using the MycoAlert kit (Lonza).

### Animal studies

Animal care and experimental procedures were performed in the animal facility accredited by the Health Science Center for Accreditation of Laboratory Animal Care and Use of the Japan Health Science Foundation. All protocols were approved by the Institutional Animal Care and Use Committee of Eisai Co., Ltd and carried out in accordance with the Animal Experimentation Regulations of Eisai Co., Ltd. For all *in vivo* studies, E7130, cetuximab (Merck Serono), trastuzumab (Chugai Pharmaceutical), or anti-mouse PD-1 antibody (BioXcell, clone RMP1-14) was administered intravenously at a dose of 0.2 mL per 20 g body weight in saline. Paclitaxel was administered intravenously at a dose of 0.2 mL per 20 g body weight in a solution consisting of 10% cremophor EL, 10% ethanol, and 4% glucose. This enabled us to administer paclitaxel to mice at up to 40 mg/kg due to low solubility of the compound. Cisplatin (Bristol-Myers Squibb) was administered intravenously at a dose of 0.2 mL of an undiluted solution per 20 g body weight. In several preclinical studies, cetuximab was intravenously injected via tail vein to mice at 2.5–10 mg/kg^32–34^, trastuzumab was intravenously injected via tail vein to mice at 10–15 mg/kg^35,36^, and cisplatin was intravenously injected via tail vein to mice at 5 mg/kg^37,38^. Our protocol that cetuximab was intravenously injected via tail vein to mice at 5 mg/kg in HSC-2 model and at 10 mg/kg in FaDu model, trastuzumab was intravenously injected via tail vein to mice at 10 mg/kg in KPL-4 xenograft model, and cisplatin was intravenously injected via tail vein to mice at 5 mg/kg in HSC-2 xenograft model were determined in reference to the previously reported protocol. In several preclinical studies, anti-mouse PD-1 antibody (BioXcell, clone RMP1-14) was injected to mice at 200 μg/mouse twice a week^39^. Our protocol that anti-mouse PD-1 antibody was administered to mice at 10 mg/kg in CT26 syngeneic model was determined in reference to the previously reported protocol because a dose of 200 μg/mouse is almost equivalent to that of 10 mg/kg. However, the antibody was intravenously injected via tail vein to mice in our study while intraperitoneal injection was used in many reported preclinical studies. Mice were obtained from Charles River. Tumour size was measured twice each week, and the formula *V* = (*d*^2^ × *D*)/2 (where *d* = minor tumour axis and *D* = major tumour axis) was used to determine the tumour volume (mm^3^, mean ± s.e.m. of individual tumour volume) or relative tumour volume to the initial tumour volume. Female BALB/C mice (BALB/cAnNCrlCrlj mice; Charles River) were used in the CT26 murine colon carcinoma cell line s.c. syngeneic model.

### HSC-2 orthotopic transplantation mouse model

Under anaesthesia by subcutaneous injection of medetomidine hydrochloride (0.15 mg/kg), midazolam (2 mg/kg) and butorphanol tartrate (2.5 mg/kg), *luciferase*-transduced HSC-2 (HSC-2-Luc) cells were inoculated into the tongue of 6-week-old (CAnN.Cg-Foxn1nu/CrlCrlj mice; Charles River) female BALB/C-nu mice (1 × 10^6^ cells in 50 μL of PBS). Seven days after transplantation, the tumour volume was analysed using the bioluminescence signal from HSC-2-Luc cells. For the bioluminescence imaging, 0.1 mL of 15 mg/mL D-luciferin (Promega) was injected intraperitoneally into the mice under 1% to 2% inhaled isoflurane anaesthesia. The bioluminescence signal was monitored using an IVIS SPECTRUM series instrument (PerkinElmer) consisting of a highly sensitive, cooled, charge-coupled device camera. Living Image software (PerkinElmer) was used to grid the imaging data and integrate the total bioluminescence signal in each region-of-interest (ROI). All bioluminescence images were acquired with a 1 second exposure. Data were analysed using total photon flux emission (photons/second) in the ROIs.

### Fluorescent-labelled antibody

Fluorescence labelling of cetuximab and anti-mouse PD-1 antibody was performed with Flamma 749 Vinylsulfone (BioActs) according to the manufacturer’s protocol. For the *in vivo* studies, fluorescent-labelled cetuximab and anti-mouse PD-1 antibody were administered at a dose of 10 mg/kg.

### Cell-based microtubule dynamics assay

A cell-based microtubule dynamics assay was conducted with the U2OS-EB3-AG osteosarcoma cell line, in which the fusion protein of EB3 (a microtubule plus end binding protein) and Azami-Green (EB3-AG) was stably expressed. U2OS-EB3-AG cells were cultured in RPMI-1640 medium containing 10% FBS, penicillin and streptomycin at 37 °C in a humidified 5% CO_2_ atmosphere. The microtubule dynamics in the live cells can be visualized as the movement of the comet-like structure of EB3-AG. U2OS-EB3-AG cells plated on glass-base culture plates (EZVIEW plate, AGC Techno Glass) were treated with E7130 at the indicated concentration, and the microtubule dynamics were monitored by time-lapse imaging using a fluorescence microscope with 60-fold magnification oil-immersion objective lens (BZ-X710, KEYENCE).

### *In vitro* antiproliferation assay

Cancer cell lines were seeded in 96-well plates. Serial dilutions of the test compounds were added one day after plating, and the viable cell number was determined 72 h after treatment by a Cell Counting Kit-8 assay (Dojindo Laboratories). The value of the wells containing cells to which only vehicle was added was defined as 100%, and the value of the wells containing no cells was defined as 0%. Three experiments were independently performed in triplicate.

### *In vitro* α-SMA induction system using normal human fibroblasts

Normal human fibroblasts BJ (ATCC) and TIG3 (JCRB) were maintained in MSCGM (Lonza) at 37 °C in a humidified atmosphere containing 5% CO_2_ and 3% O_2_. In co-culture experiments, 1 × 10^4^ normal human fibroblasts and 5 × 10^3^ FaDu cells were mixed in 200 μL of DMEM containing 0.1% FBS and plated in each well of 96-well culture plates and cultured for 3 days. If indicated, A83-01, a potent selective inhibitor of TGF-β-R, was added at a concentration of 1 μmol/L during the culture period. To examine the effects of E7130 on the TGF-β-induced expression of α-SMA, normal human fibroblasts in 100 μL of DMEM containing 0.1% FBS were seeded at 2 × 10^4^ cells per well in 96-well plates. The next day, TGF-β (final concentration of 1 ng/mL) and E7130 at the indicated concentrations in the same medium were added to each well so that the total volume was 200 μL. The treated cells were cultured for an additional 2 days before being subjected to immunocytochemical analysis.

### Immunohistochemical analysis

Microvessel density in the xenograft tumour samples was analysed by immunohistochemistry using the anti-CD31 antibody (DIA-310, Dianova). The area of α-SMA-positive CAFs in the xenograft tumour samples was analysed by immunohistochemistry using an anti-α-SMA antibody conjugated with alkaline phosphatase (A5691, Sigma). The area of Ki67-positive cancer cells in the xenograft tumour samples was analysed by immunohistochemistry using an anti-Reticular Fibroblasts and Reticular Fibres (ER-TR7) antibody (ab51824; Abcam). The overall stroma area in the xenograft tumour samples was analysed by immunohistochemistry using an anti-Ki-67 antibody (#12202; Cell Signaling Technology). Immunostained slides were scanned using the Vectra 2 Automated Slide Imaging System (Perkin Elmer), and quantification of the whole tumour was performed using the inForm 2 software (Perkin Elmer). The area of the tumour region was measured by assessing the haematoxylin-stained area.

### Immunocytochemical analysis

After the indicated culture periods, the plated cells were fixed for 10 minutes at room temperature in 4% paraformaldehyde diluted with PBS, and then, they were permeabilized with cold methanol at −20 °C for 10 minutes and incubated with the primary antibodies. For immunofluorescence, Alexa Fluor 647 goat anti-rabbit and Alexa Fluor 555 goat anti-mouse were used as secondary antibodies (Life Technologies), and DAPI was used for nuclear staining. The primary antibodies used and their incubation conditions are listed in Supplementary Table S1. Immunofluorescence images were obtained by a BZ-X700 all-in-one fluorescence microscope system (KEYENCE). The quantification was carried out with four-fold magnified images of the central region of the stained wells using Hybrid cell count software (KEYENCE). The expression level of α-SMA was calculated by dividing the integrated luminance value of α-SMA by the number of nuclei stained with DAPI (cell number).

### Western blot analysis

Whole cell lysates were separated using SDS–PAGE and transferred to PVDF membranes. The blots were blocked with 10% non-fat dry milk at room temperature for 1 hour, and incubated overnight at 4 °C with the corresponding primary antibodies, followed by incubation with HRP-conjugated secondary antibodies (#7074, Cell Signaling Technology) at room temperature for 2 hours. The blots on the membranes were developed with Lumi-Light PLUS Western Blotting Substrate (#12015196001, Roche). The following primary antibodies were used: anti-smad antibody (#8685, Cell Signaling Technology); anti-phospho-smad antibody (#8828, Cell Signaling Technology); anti-S6 antibody (#2217, Cell Signaling Technology); anti-phospho-S6 antibody (#4857, Cell Signaling Technology); anti-Akt antibody (#4691, Cell Signaling Technology); anti-phospho-Akt antibody (#4060, Cell Signaling Technology); anti-beta-actin antibody (#4967, Cell Signaling Technology). Densitometric measurements of the protein of interest were carried out using Fusion CAPT Advance software version 17.03 (Vilber Lourmat).

## Supplementary information

Supplementary Information

Supplementary Video 1

Supplementary Video 2

## Data Availability

The datasets generated during and/or analysed during the current study are available from the corresponding author on reasonable request.
