# Supplementary material for: A landmark in drug discovery based on complex natural product synthesis
Source: Sci Rep. 2019 Jun 17;9:8656. doi: 10.1038/s41598-019-45001-9 (PMC6572832; doi:10.1038/s41598-019-45001-9)
Supplement: Supplementary file 1 — Supplementary Information [file 41598_2019_45001_MOESM1_ESM.pdf]

Supplementary Information

Title:

A landmark in drug discovery based on complex natural product synthesis

Authors:

Satoshi Kawano[1]*, Ken Ito[1], Kenzo Yahata[2], Kazunobu Kira[1], Takanori Abe[1], Tsuyoshi Akagi[1], Makoto Asano[1], Kentaro Iso[1], Yuki Sato[1], Fumiyoshi Matsuura[3], Isao Ohashi[1], Yasunobu Matsumoto[1], Minetaka Isomura[1], Takeo Sasaki[1], Takashi Fukuyama[1], Yusuke Miyashita[4], Yosuke Kaburagi[1], Akira Yokoi[1], Osamu Asano[1], Takashi Owa[5] & Yoshito Kishi[2]*

[1]Eisai Co., Ltd., Tokodai, Tsukuba-shi, Ibaraki, Japan.
[2]Harvard University, Cambridge, Massachusetts, USA.
[3]Eisai Co., Ltd., Koishikawa, Bunkyo-ku, Tokyo, Japan.
[4]Eisai Co., Ltd., Sunayama, Kamisu-shi, Ibaraki, Japan.
[5]Eisai Inc., Woodcliff Lake, New Jersey, USA.

*Correspondence to:
s-kawano@hhc.eisai.co.jp (S.K.); kishi@chemistry.harvard.edu (Y.Ki)

Contents

1) Supplementary methods
2) Supplementary Figure S1
3) Supplementary Figure S2
4) Supplementary Figure S3
5) Supplementary Figure S4
6) Supplementary Figure S5
7) Supplementary Figure S6
8) Supplementary Figure S7
9) Supplementary Figure S8
10) Supplementary Table S1
11) Supplementary Video 1
12) Supplementary Video 2

Supplementary methods

***In vitro* tubulin polymerization assay**

A tubulin polymerization assay kit was purchased from Cytoskeleton. The kit contained 1 bottle of lyophilized tubulin protein purified from porcine brain, 3 tubes of lyophilized GTP, 2 bottles of lyophilized assay buffer, and 1 bottle of tubulin glycerol buffer. Assay buffer was prepared by dissolving the contents in 10 mL of deionized and sterilized water. This solution contained 80 mmol/L piperazine-*N,N'*-bis[2-ethanesulfonic acid] sesquisodium salt, 2.0 mmol/L magnesium chloride, 0.5 mmol/L ethylene glycol-bis(2-amino-ethyl ether) *N,N,N',N'*-tetra-acetic acid, and 10 µmol/L fluorescent reporter at pH 6.9. The buffer was stored at –70 °C until use. The tubulin glycerol buffer consisted of 80 mmol/L piperazine-*N,N'*-bis[2-ethanesulfonic acid] sesquisodium salt, 2.0 mmol/L magnesium chloride, 0.5 mmol/L ethylene glycol-bis(2-amino-ethyl ether) *N,N,N',N'*-tetra-acetic acid, and 60% v/v glycerol at pH 6.9. This buffer was stored at 4 °C until use. The GTP stock solution was prepared by dissolving the contents of each tube in 100 µL of deionized and sterilized water to achieve a concentration of 100 mmol/L GTP. Aliquots of this stock were stored at –70 °C until use. The tubulin stock solution (10 mg/mL) was prepared by dissolving the tubulin powder in 1.1 mL of the mixture of assay buffer and GTP stock solution (100:1, v/v). Aliquots were frozen in liquid nitrogen and then stored at –70 °C until use. In the tubulin polymerization assay, the reaction mixture was prepared by mixing 820 µL of assay buffer, 17.6 µL of GTP stock solution, and 600 µL of tubulin glycerol buffer. The reaction mixture (1015 µL) was combined with 240 µL of the tubulin stock solution. This solution was referred to as the tubulin reaction mixture and was used for the measurement of the test and control wells. A tubulin-free reaction mixture was prepared by mixing 89.85 µL of reaction mixture and 21.25 µL of assay buffer, and this mixture was used for the blank wells. Aliquots of E7130 solution (6.25–100 µmol/L; final concentrations of 0.625–10 µmol/L) or vehicle (5 µL) was added to the individual wells of a 96-well half-area microtiter plate. The tubulin reaction mixture or tubulin-free reaction mixture (45 µL) was added to each well of the plate. Fluorescence emission at 460 nm (excitation wavelength of 360 nm) was measured every 2 minutes for 90 minutes using a SpectraMax® M5e microplate reader (Molecular Devices). Tubulin polymerization was followed by fluorescence enhancement due to the incorporation of a fluorescence reporter into the microtubules during polymerization. The assay was performed in duplicate.

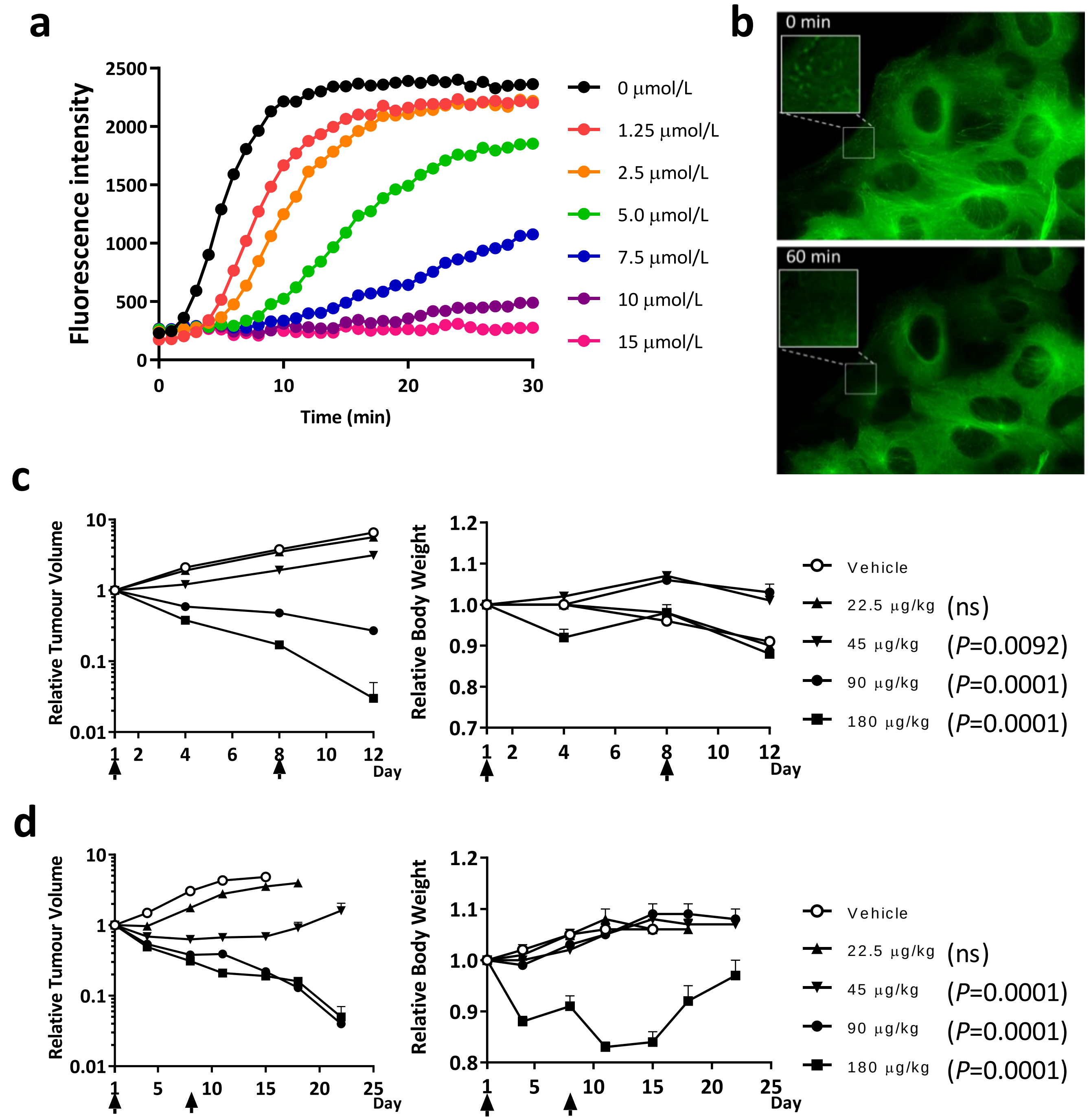

Supplementary Figure S1: Biochemical, cellular, and *in vivo* mechanistic activity of E7130

a, Tubulin dynamics inhibitory effect of E7130. Tubulin polymerization could be monitored by fluorescence enhancement due to the incorporation of a fluorescence reporter into the microtubules during polymerization. b, Effect of E7130 against the U2OS osteosarcoma cell line, in which the fusion protein of EB3 (a microtubule plus end binding protein) and Azami-Green (EB3-AG) are expressed. By treatment with E7130 for 60 minutes at 0.5 nmol/L, which was its IC_{50} value for the antiproliferative activity in cells, the comet-like structures became hard to detect. Time-lapse movies before and 60 minutes after the treatment with E7130 are available in Supplementary Video 1 and 2, respectively. c, d, Effect of E7130 administered on day 1 and day 8 on the relative tumour volume and relative body weight of the subcutaneous KPL-4 (c) and OSC-19 (d) xenograft models. In the studies, thirteen days (KPL-4) or four days (OSC-19) after the cell inoculation subcutaneously in the right flank of Balb/C-nu mice, 40 mice were selected based on their tumour volumes and shapes of tumours, and were randomly allocated into 5 groups (day 1). The mean tumour volumes of mice assigned to the groups on day 1 were 108.0 mm^3 (KPL-4) and 226.3 mm^3 (OSC-19). The mean relative values to those of day 1 ± s.e.m. are shown (n=8). *P* values are from Dunnett's multiple comparison test versus vehicle control on day 12 (KPL-4) and day 15 (OSC-19).

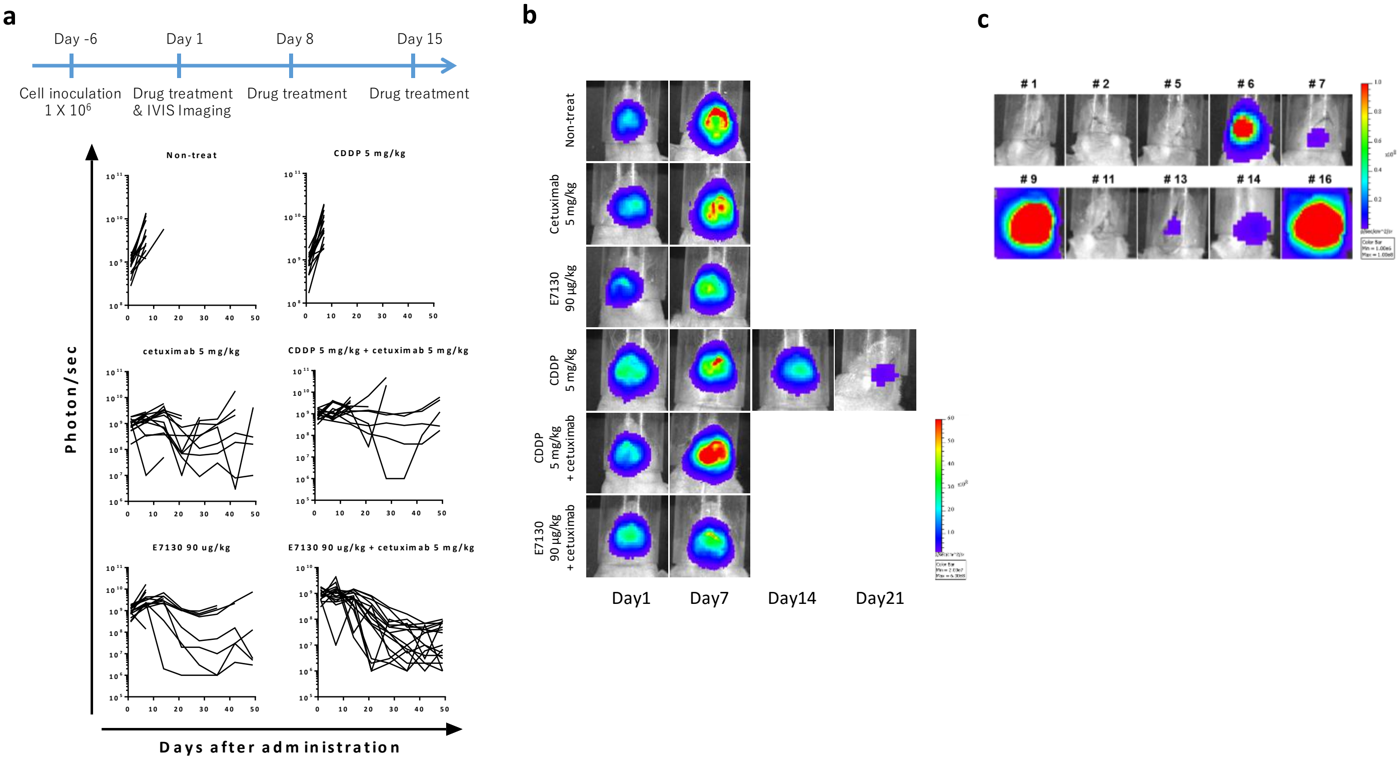

Supplementary Figure S2: The amount of *luciferase*-transduced HSC-2 was analysed using an In Vivo Imaging System (IVIS)

a, Data show the bioluminescence levels in tongue of each mouse. b, Representative bioluminescence image. c, The amount of *luciferase*-transduced HSC-2 was analysed using an IVIS. Bioluminescence images of 10 survived mice from the E7130+cetuximab combination group on day 100.

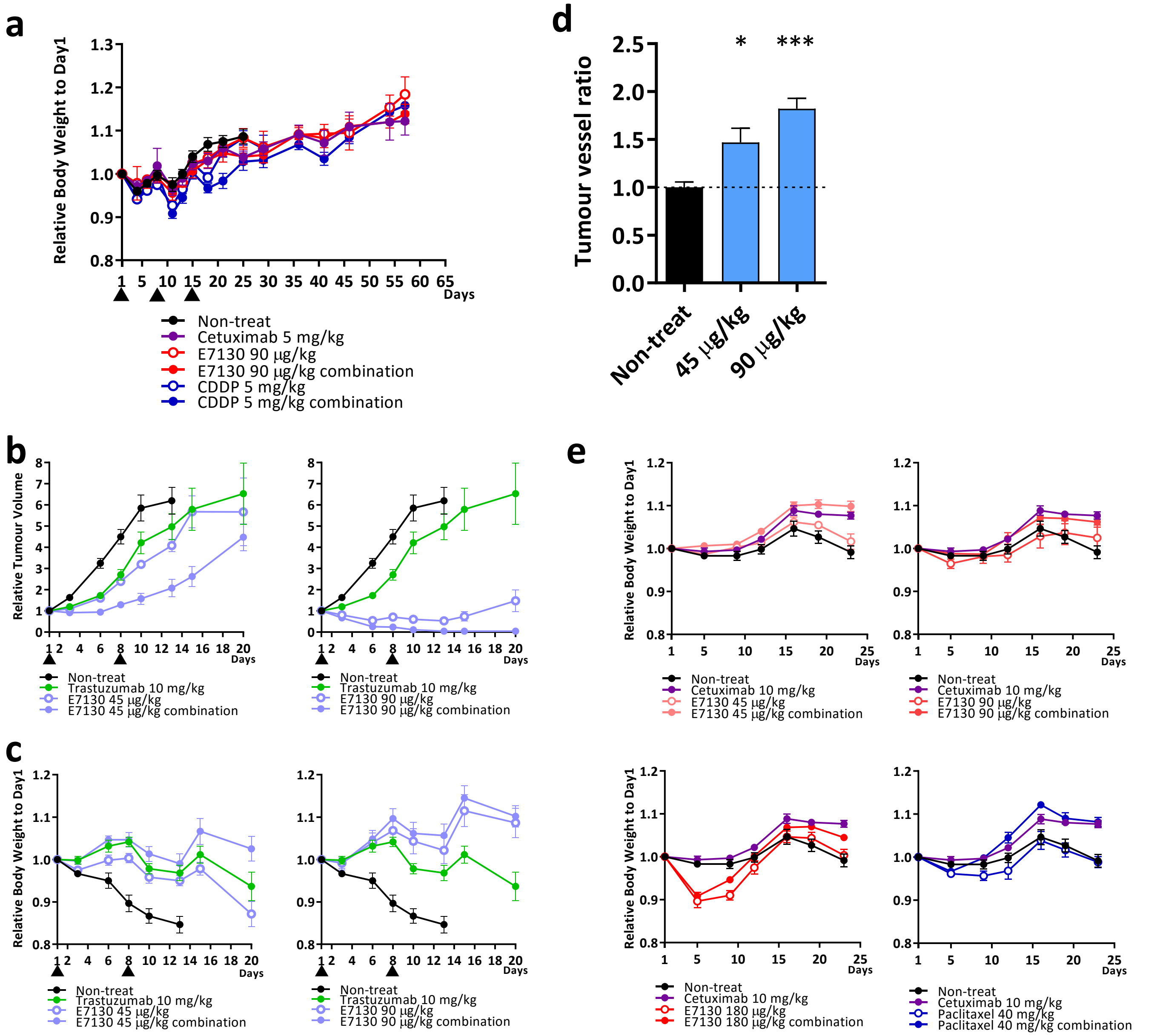

Supplementary Figure S3: Effect of E7130 in studies using several xenograft models

a, Effect of E7130 on the relative body weight of the subcutaneous HSC-2 xenograft model. The mean relative body weight to that on day 1 ± s.e.m. is shown (n=6). b, c, Effect of indicated administration on day 1 and day 8 on the relative tumour volume (b) and relative body weight (c) of the subcutaneous KPL-4 xenograft model. In this study, fourteen days after the cell inoculation subcutaneously in the right flank of Balb/C-nu mice, 36 mice were selected based on their tumour volumes and shapes of tumours, and were randomly allocated into 6 groups (day 1). The mean tumour volume of mice assigned to the groups on day 1 were 200.0 mm^3 (KPL-4). The mean relative values to those on day 1 ± s.e.m. are shown (n=6). d, KPL-4 xenografts were collected 5 days after the administration of E7130 (45 and 90 μg/kg, intravenously). Data show the mean tumour vessel ratios of those from the treatment groups to those of the non-treated group ± s.e.m. (n=5). * *P*=0.0210, *** *P*=0.0004 versus non-treated group (Dunnett's multiple comparison test). e, Effect of indicated administration on day 1 on the relative body weight of the subcutaneous FaDu xenograft model. The mean relative body weight to that on day 1 ± s.e.m. is shown (n=6).

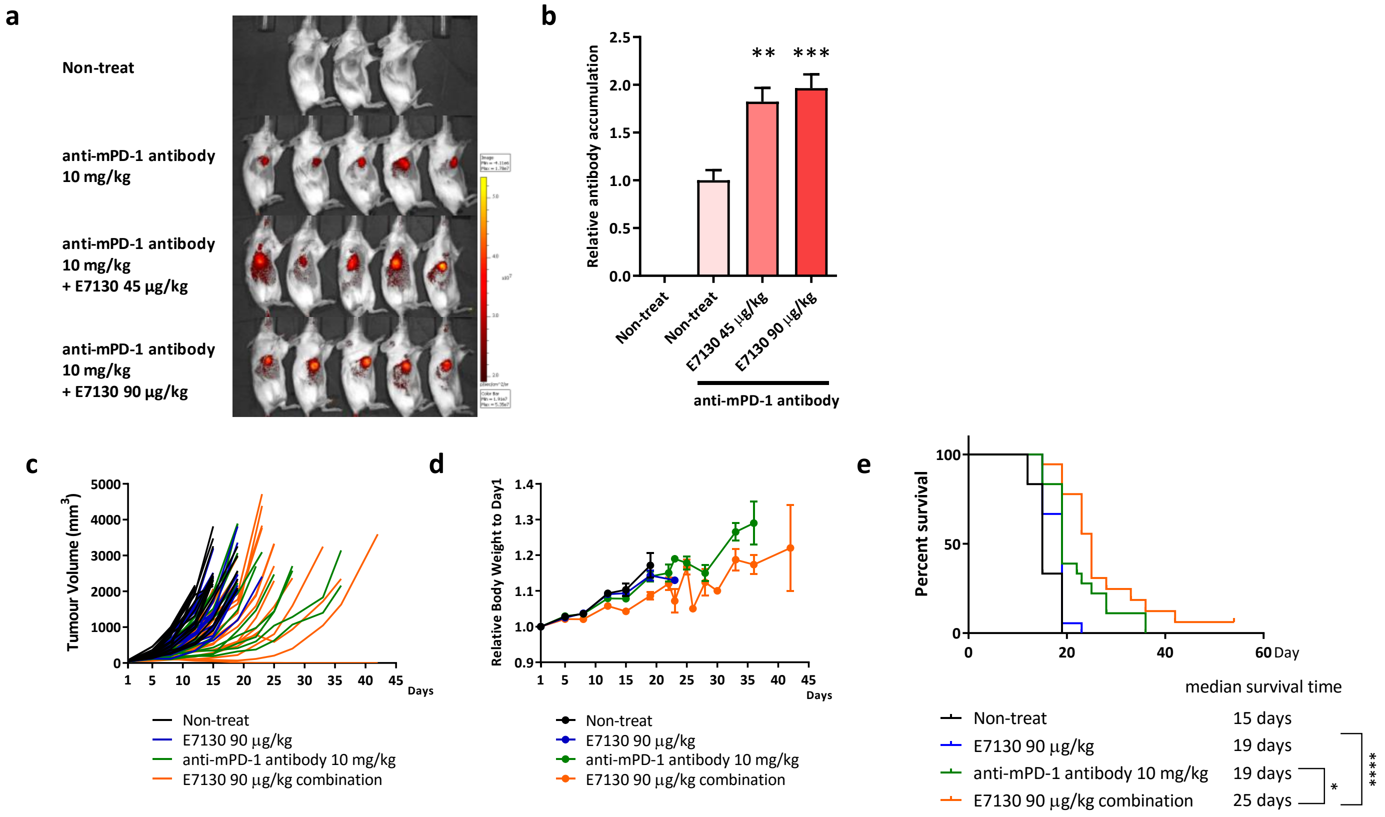

Supplementary Figure S4: Effect of E7130 in combination with anti-mouse PD-1 antibody

a, b, Fluorescent-labelled anti-mouse PD-1 antibody was intravenously injected with or without E7130, and the accumulation of fluorescent-labelled antibody was analysed using the IVIS 5 days after the administration. Fluorescence images (a) and the quantification analysis data (b). The graph shows the mean ratios of the fluorescent-labelled anti-mouse PD-1 antibody accumulation of those from the E7130+fluorescent-labelled anti-mouse PD-1 combination groups to those of the fluorescent-labelled anti-mouse PD-1 antibody mono-administration group ± s.e.m. (n=5). ** *P*=0.0017, *** *P*=0.0005 versus fluorescent-labelled anti-mouse PD-1 antibody mono-administration (Dunnett's multiple comparison test). c, d, Effect of the indicated administration on the tumour volume (c) and relative body weight (d) of the CT26 murine colon carcinoma cell line s.c. syngeneic model. E7130 were administrated at days 1, 8, 15, and 22, and anti-mouse PD-1 antibody was administered twice a week for four weeks. Individual tumour volume (c) and mean body weight relative to that on day 1 ± s.e.m. (d) are shown (n=18). e, Evaluation of survival patterns in tumour-bearing mice was performed using the Kaplan–Meier method, and the results are ranked according to the Gehan-Breslow-Wilcoxon test. * *P*=0.0412, **** *P*<0.0001. Mice with tumours >2000 mm^3 or appearance of moribund at any time were defined as endpoint.

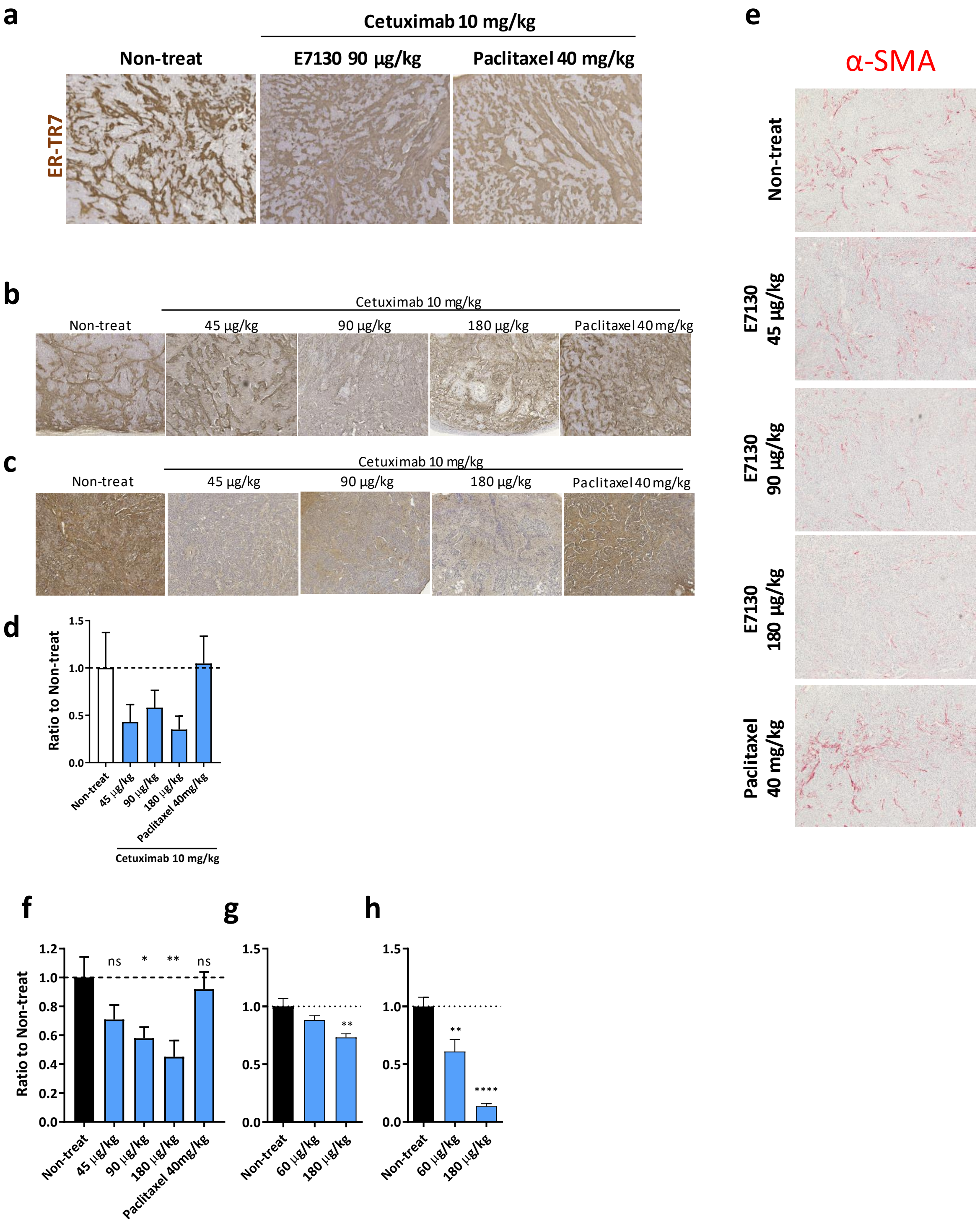

Supplementary Figure S5: E7130 showed an anti-CAF effect

a-d, FaDu xenografts were collected 10 days after the administration as indicated. The areas of pan-fibroblast (a), tenascin-C (b), and EDA-fibronectin (c, d) were analysed by immunohistochemistry using an anti-pan-fibroblast antibody (ER-TR7), anti-tenascin-C antibody, and anti-EDA-fibronectin antibody, respectively. Representative images are shown (a-c). Data show the mean area of EDA-fibronectin in the treatment groups to that of the non-treated group ± s.e.m. (n=5) (d). e, f, FaDu xenografts were collected 2 days after the administration as indicated. The area of α-SMA-positive CAFs was analysed by immunohistochemistry using the anti-α-SMA antibody. * *P*=0.0420, ** *P*=0.0080 versus the non-treated group (Dunnett's multiple comparison test). Representative images are shown in e. g, h, HSC-2 (g) and OSC-19 (h) xenografts were collected 2 days and 5 days after the intravenously administration of E7130, respectively. (g) ** *P*=0.0038, (h) ** *P*=0.0067, **** *P*=0.0001 versus the non-treated group (Dunnett's multiple comparison test).

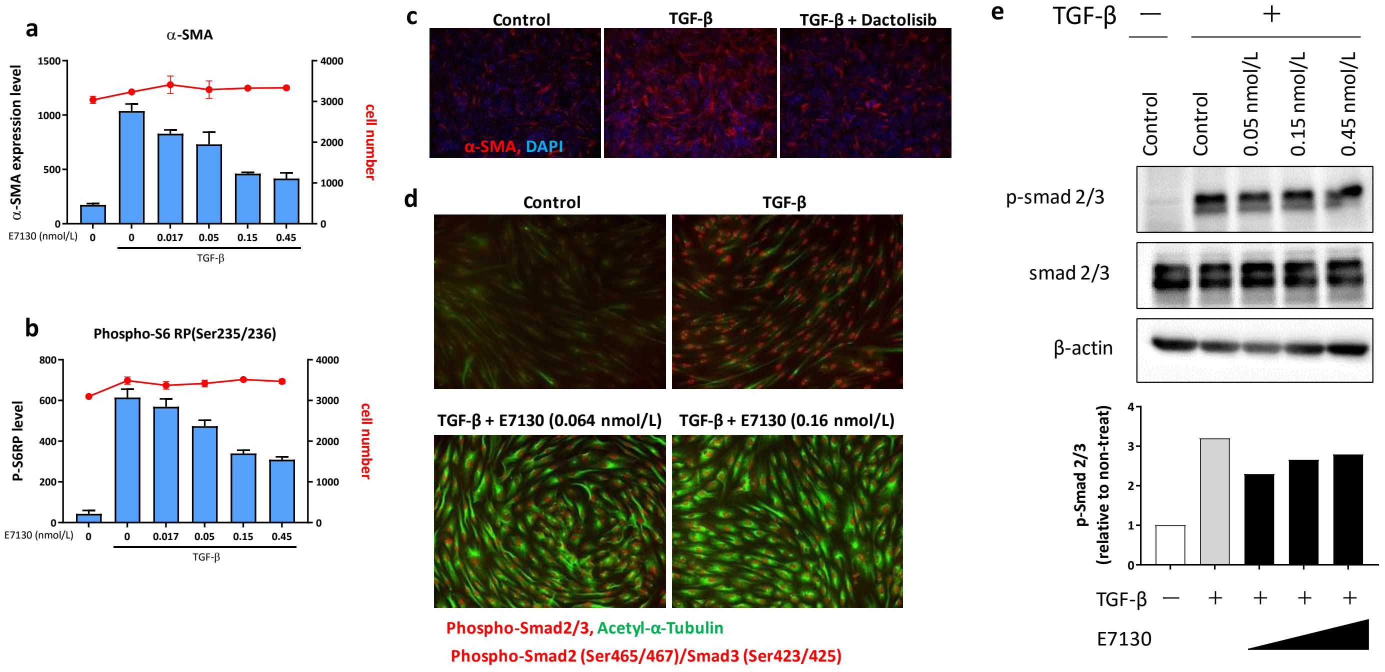

Supplementary Figure S6: E7130 interfered with α-SMA induction by TGF-β in BJ cells

a, b, Quantification of immunofluorescence images of BJ cells treated with TGF-β (1 ng/mL) and the indicated concentrations of E7130 for α-SMA (a), Phospho-S6-ribosomal protein (Ser235/236) (b). c, Immunofluorescence analysis of BJ cells treated with TGF-β (1 ng/mL) in the absence or presence of dactolisib, a phosphatidylinositol 3 kinase inhibitor (100 nmol/L). d, Immunofluorescence analysis of phosphorylated Smad2/3 in BJ cells pretreated with E7130 (72 hours) after 30 minutes of stimulation with TGF-β (1 ng/mL). Acetylated-α-tubulin was co-stained to confirm the activity of E7130. e, Western blot analysis of Smad2/3 in BJ cells treated with TGF-β (1 ng/mL) and the indicated concentrations of E7130. The lens magnification used was ✕4 (c) or ✕40 (d).

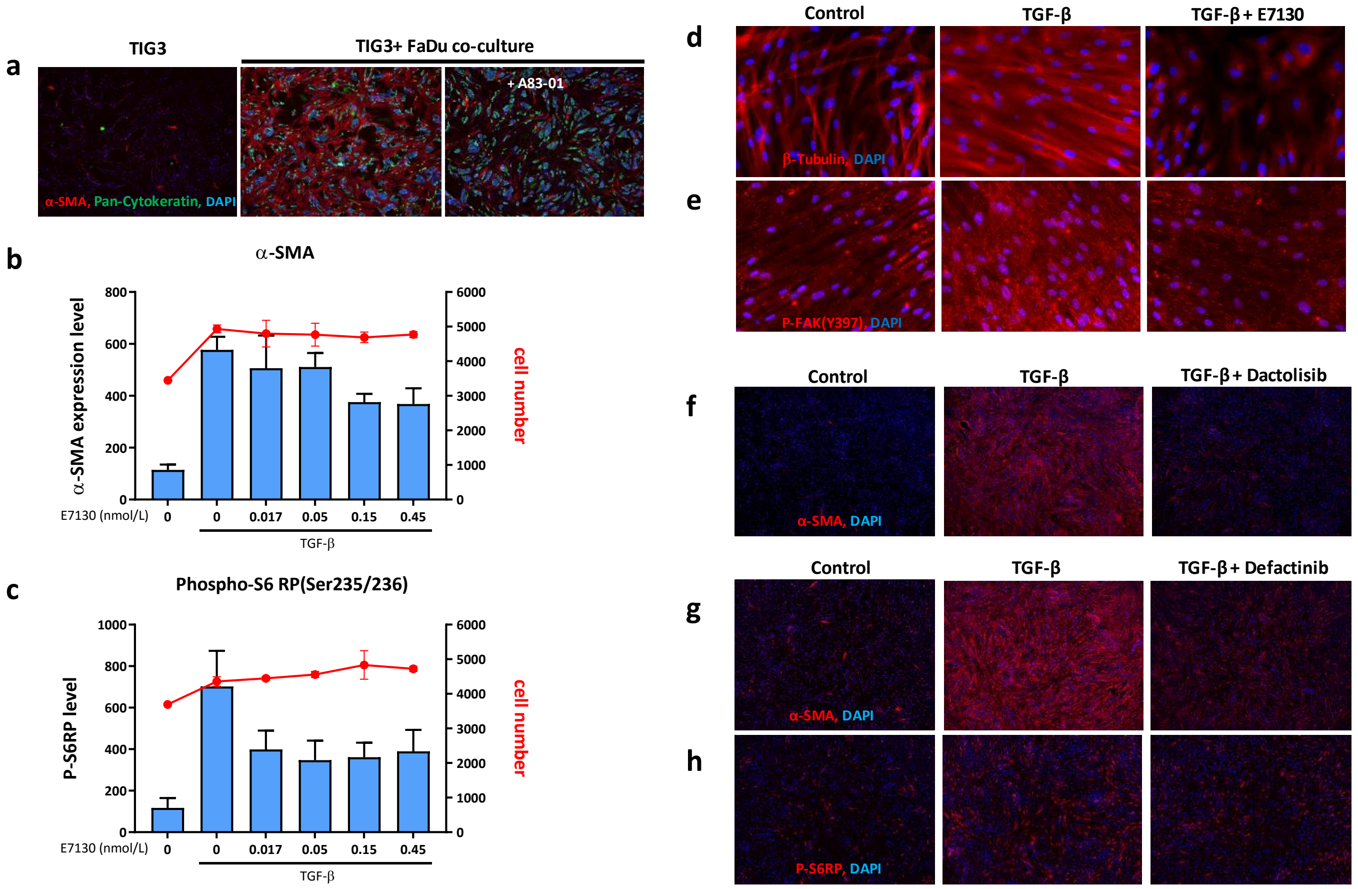

Supplementary Figure S7: E7130 interfered with α-SMA induction by TGF-β in TIG3 cells

a, Immunofluorescence staining of α-SMA (red colour) and pan-cytokeratin (green colour) in TIG3 co-cultured with FaDu in the absence or presence of A83-01, an FAK inhibitor. b, c, Quantification of immunofluorescence images of TIG3 cells treated with TGF-β (1 ng/mL) and the indicated concentrations of E7130 for α-SMA (b), Phospho-S6-ribosomal protein (Ser235/236) (c). d, e, Immunofluorescence analysis of TIG3 cells treated with TGF-β (1 ng/mL) in the absence or presence of E7130 for β-tubulin (d), Phospho-FAK (Tyr397) (e). f, Immunofluorescence analysis of TIG3 cells treated with TGF-β (1 ng/mL) in the absence or presence of dactolisib (100 nmol/L) for α-SMA. g, h, Immunofluorescence analysis of TIG3 cells treated with TGF-β (1 ng/mL) in the absence or presence of defactinib (3 μmol/L) for α-SMA (g), Phospho-S6-ribosomal protein (Ser235/236) (h). The lens magnification was ×4 (a, f-h) or ×40 (d, e).

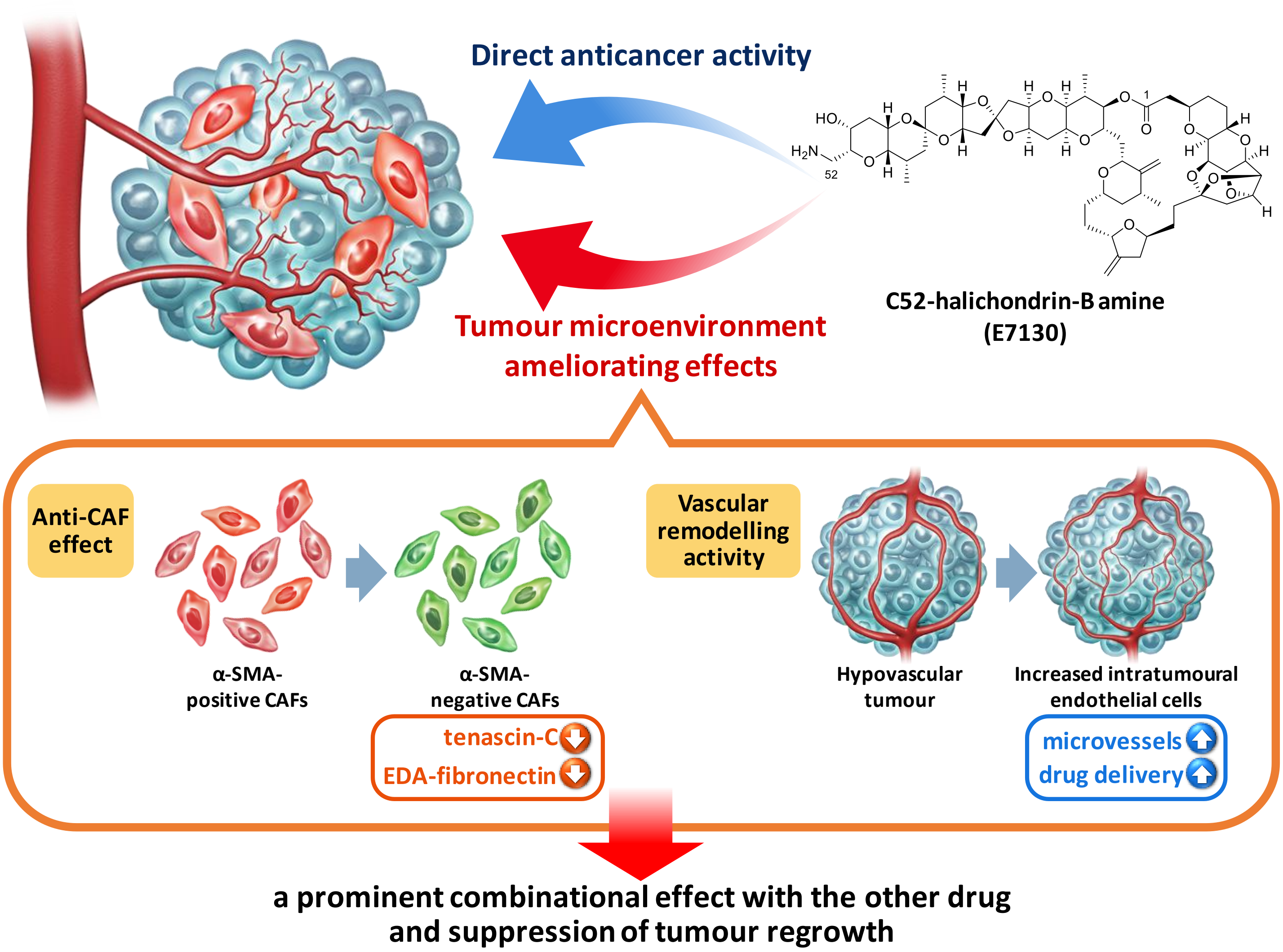

Supplementary Figure S8: Schematic illustration of direct anticancer activity and effects on the tumour microenvironment

Antibody	Supplier	Dilution	Incubation
Anti-pan-cytokeratin (AE1/AE3; mouse monoclonal)	Dako (Glostrup, Denmark)	1:100	overnight at 4 °C
Anti-α-SMA (ab5694; rabbit polyclonal)	Abcam (Cambridge, UK)	1:200	overnight at 4 °C
Anti-phospho-smad2 (Ser465/467)/smad3 (Ser423/425) (D27F4; rabbit monoclonal)	Cell Signaling Technology (Danvers, USA)	1:200	overnight at 4 °C
Anti-acetyl-α-tubulin (Lys40) (D20G3 ; rabbit monoclonal)	Cell Signaling Technology (Danvers, USA)	1:800	overnight at 4 °C
Anti-β-tubulin (9F3; rabbit monoclonal)	Cell Signaling Technology (Danvers, USA)	1:100	overnight at 4 °C
Anti-FAK (phospho Y397) (EP2160Y; rabbit monoclonal)	Abcam (Cambridge, UK)	1:200	overnight at 4 °C

Supplementary Table S1: List of antibodies used for immunofluorescence

Supplementary Video 1: Time-lapse movie of the U2OS osteosarcoma cell line without treatment
Time-lapse movies of the U2OS osteosarcoma cell line in which the fusion protein of EB3 (a microtubule plus end binding protein) and Azami-Green (EB3-AG) are expressed, without the treatment with E7130.

Supplementary Video 2: Time-lapse movie of the U2OS osteosarcoma cell line after treatment with E7130
Effect of E7130 against the U2OS osteosarcoma cell line, in which the fusion protein of EB3 (a microtubule plus end binding protein) and Azami-Green (EB3-AG) are expressed. By treatment with E7130 for 60 minutes at 0.5 nmol/L, which was its IC_{50} value for the antiproliferative activity in cells, the comet-like structures became hard to detect.
